# Sex-related differences in single- and multi-arterial coronary artery bypass grafting: Insights from the Netherlands Heart Registration

**DOI:** 10.1371/journal.pone.0336035

**Published:** 2025-12-31

**Authors:** Sophie H.Q. Beukers, Edgar J. Daeter, Lineke Derks, Geoffrey T.L. Kloppenburg, on behalf of the Cardiothoracic Surgery Registration Committee of the Netherlands Heart Registration

**Affiliations:** 1 Department of Cardiothoracic Surgery, St. Antonius Hospital, Nieuwegein, the Netherlands; 2 Netherlands Heart Registration, Utrecht, the Netherlands; James Cook University Hospital, UNITED KINGDOM OF GREAT BRITAIN AND NORTHERN IRELAND

## Abstract

Women are known to suffer from increased mortality and major adverse cardiac events rates after coronary artery bypass grafting compared to men. This study gives an overview of sex-disparities in grafting strategy and outcome of coronary artery bypass grafting in the Netherlands, and compares survival of the younger population undergoing multi-arterial grafting. Data were gathered retrospectively from the Netherlands Heart Registration database. Patients undergoing isolated after coronary artery bypass grafting were divided into groups treated with single or multi-arterial grafting. Using risk adjusted cox proportional hazard regression analysis, the effect of sex on the revascularization strategy and postoperative outcomes was assessed. Secondary analysis was conducted on a subset of patients aged 70 years or younger at baseline. The study included 51137 patients, of which 19.1% was female. When compared to men, women were older and suffered from more comorbidity. Female sex was independently associated with less multi-arterial grafting. While multi-arterial grafting led to a lower repeat revascularization rate in men (p = 0.022), this was not the case in women. Cox regression analysis did not independently associate the female sex with inferior survival. In the younger population, a survival benefit was observed after multi-arterial grafting, to the point where survival did not differ between sexes. Women receive fewer arterial grafts as opposed to men. In a younger patient population, the survival difference between sexes disappears when patients are treated with multi-arterial grafting.

## Introduction

Controversies regarding the choice of graft material in patients undergoing coronary artery bypass grafting (CABG) persist to date. Especially the optimal graft strategy for women, who present at older age and with more complex disease due to comorbidity, has not been determined. After CABG, women suffer from increased mortality and complication rates as compared to men, of which the cause is unclear.

In the perioperative period, the female sex is independently associated with increased mortality [1–3]. Women more often require blood transfusions and suffer from a higher major adverse cardiac and cerebrovascular events (MACCE) rate [2–4]. After discharge, women have an increased risk of re-admittance for recurring angina or congestive heart failure [1,5]. In long-term follow-up, increased mortality is observed in women as opposed to men, although this might not pertain to the elderly population of after propensity score matching [1–3,6].

Notable differences in surgical revascularization strategy have been demonstrated between men and women. Women receive fewer arterial grafts and anastomoses, while they could receive benefits similar to men of multi- or total arterial grafting (MAG or TAG) [4,7–9]. Incomplete revascularization rates are higher in women [7]. Studies regarding graft patency in women reveal contrasting results. Reported graft occlusion rates of the radial artery (RA) and bilateral internal thoracic arteries (BITA) are similar, but data on saphenous vein graft patency in women is ambiguous [10–13].

Numerous studies have demonstrated superior survival and freedom of MACCE or graft failure after MAG or TAG over the use of a single arterial graft (SAG) [14–17]. An extensive meta-analysis and one of the largest randomized controlled trial performed to date on this topic unexpectedly failed to do so [2,18]. Given the substantial underrepresentation of women in trials, uncertainty whether women are subjected to suboptimal treatment remains [15–18]. The aim of this study is to assess differences in baseline and outcomes between sexes in undergoing isolated CABG using SAG or MAG in the Netherlands. Subsequently in the population aged 70 years or younger, survival after MAG will be compared between sexes, as this is the population that should gain a long-term survival benefit from MAG. Secondly, the effect of sex on the revascularization strategy is determined, to clarify if and why women are less often treated with MAG.

## Materials and methods

### Data source

The database of The Netherlands Heart Registration (NHR) is a prospective quality registry containing data of all patients undergoing cardio-thoracic surgery in the Netherlands. The dataset is comprised of an anonymized patient identification number, all variables included in EuroSCORE II and several variables on operative technique and postoperative outcome. Mortality data were obtained from the regional municipal administration registration of the Netherlands (Basisregistratie Personen). The procedure of data collection and quality assessment have previously been described [19,20]. The study protocol and requested dataset were approved by the NHR, clinical registry number 2022−003. Access to data was provided in April 2023. The Medical Research Ethics Committees United (W19.270) waived the need for informed consent for use of the anonymized data of the NHR. The study complied with the principles of Declaration of Helsinki.

### Population

A nationwide retrospective database study was conducted on patients undergoing isolated CABG using one or more arterial grafts in the period January 2013 to December 2020. Patients with a history of cardiac surgery were excluded.

### Subgroups

Patients were divided into two groups based on the revascularization strategy. These groups were SAG – patients receiving one arterial graft – and MAG. MAG was defined as the use of two or more of the following arterial grafts: LITA, right internal thoracic artery (RITA), RA, gastroepiploic artery (GEA) or inferior epigastric artery. Patients in both groups might also have one or more additional venous grafts. Secondary analysis was conducted for patients aged 70 years or younger.

### Research objectives

Primary endpoint was survival comparison between sexes after SAG and MAG. Secondary endpoints were the effect of sex on the distribution of MAG, freedom from repeat revascularization; either percutaneous or surgical, and deep sternal wound infection (DSWI) rate, with a secondary analysis on DSWI rate after use of BITA versus a single internal thoracic artery (SITA).

### Variables

Preoperative variables were composed of age, sex, height and weight, serum creatinine levels, comorbidities, left ventricular ejection fraction (LVEF), New York Heart Association (NYHA) functional class, European System for Cardiac Operative Risk Evaluation (EuroSCORE) II and unstable angina or critical preoperative state at the time of admission. Comorbidities included chronic pulmonary disease, diabetes mellitus, extracardiac arteriopathy, neurological dysfunction, prior cerebrovascular accident (CVA), dialysis and recent myocardial infarction (MI); defined as MI within 90 days or less before surgery. Kidney failure was defined as estimated glomerular filtration rate <60 mL/min or patient receiving dialysis.

Operative variables consisted of urgency of the procedure (emergent or non-emergent), graft material and number of distal anastomoses. For patients undergoing CABG with aortic cross-clamping, cross-clamp-time per distal anastomosis was calculated by dividing the cross-clamp time in minutes by the number of distal anastomoses. Emergent surgery was defined as surgery that must be performed on the same date as acceptance for surgery. Non-emergent surgery was defined as planned surgery during the current admission of a patient or surgery for which the patient was routinely admitted.

### Outcomes

Short-term complications occurring within 30 days postoperative were assessed, including DSWI, resternotomy, major and minor vascular complications and repeat cardiac surgery. Complications during hospital admission included CVA and death. Definitions of complications can be found in S1 File. Mortality status and time to death or follow-up and occurrence of and time to repeat revascularization (repeat CABG or PCI) were analyzed.

### Statistics

Continues values are expressed as mean ± standard deviation, or median [interquartile range Q1-Q3], depending on distribution. Normal distribution was tested using the Quantile-Quantile plot and Kolgomorov-Smirnov test. Continuous variables were compared using the independent sample t-test when normally distributed, or using the Mann-Whitney U test when non-normally distributed.

Categorical values were compared between subgroups using the Chi square test. Overall survival and freedom from repeat revascularization rates were assessed using the Kaplan-Meier method. By means of a Log-rank test, curves were compared.

Risk adjusted Cox proportional hazards regression analysis was conducted to determine the effect of sex on survival and repeat revascularization. Results are reported using odds ratios (OR) or hazard ratios (HR) with corresponding95% confidence intervals (CI) and p-values. Baseline determinants of mortality and revascularization mode (SAG or MAG) were initially assessed using univariable regression, including age, height, unstable angina and comorbidities. Variables demonstrating statistical significance were subsequently entered in a stepwise manner into a multivariate model, retaining only those with significant independent associations. A second Cox regression was performed to assess effect of interactions between sex, age and MAG.

Binary logistic regression was used to assess the association between baseline characteristics and the likelihood of MAG among men and women aged 70 years or younger. All baseline variables and the procedural urgency were included, except for those with evident clinical collinearity, such as unstable angina and critical preoperative state when using urgency of the procedure. To assess the effect of relative height within each sex, distributional differences between sexes were accounted for by dichotomizing height at the sex-specific median using the average body height values reported by the Central Bureau of Statistics of the Netherlands.

To enable comparison of short-term complications between sexes without interference of baseline differences, propensity score matching was performed. Propensity scores were estimated using a logistic regression model which included age, body mass index, diabetes mellitus, kidney dysfunction, LVEF, chronic pulmonary disease, extracardiac arteriopathy, neurological dysfunction, recent MI, prior CVA and procedural urgency as predictors and sex as the dependent variable. One-to-one nearest-neighbor matching without replacement was performed using the MatchIt package in R with a caliper width of 0.2 standard deviations of the logit of the propensity score. Covariate balance before and after matching was evaluated using standardized mean differences (SMD). An SMD < 0.10 was considered a negligible imbalance. Balance diagnostics and Love plots were generated using the cobalt package.

A full case analysis was conducted and an alpha level of 0.05 or less was considered statistically significant. All tests were performed using SPSS (IBM Corp. Released 2017. IBM SPSS Statistics for Windows, Version 28.0. Armonk, NY: IBM Corp.). Propensity score matching and data visualization were conducted using RStudio (Posit Software, PBC. RStudio/2024, version 4.4.3).

## Results

### Baseline characteristics

A total of 51137 patients were included in the analysis, of which 19.1% was female. The majority of patients were in NYHA functional class I or II and had a normal LVEF. At baseline, women were older in comparison to men (68.7 vs 65.9 years, p < 0.001), suffered from more comorbidities and presented more often with unstable angina (Table 1). Contrary to men, women had a higher EuroSCORE II, in both the general population (1.91 ± 2.32 vs 2.94 ± 3.32, p < 0.001) and the population undergoing MAG (1.27 ± 1.29 vs 2.06 ± 2.12, p < 0.001).

**Table 1:** Baseline characteristics of the general population. Mean ± standard deviation, median [range], number (percentage).

	Men (n = 41379)	Women (n = 9758)	p-value
Age (years)	65.9 ± 9.5	68.7 ± 9.4	**<0.001**
BMI (kg/m^2^)	27.6 ± 4.0	27.8 ± 5.4	**<0.001**
LVEF			**<0.001**
≥50%	29562 (71.4)	7419 (76.0)	
30-49%	9572 (23.1)	1876 (19.2)	
<30%	2281 (5.5)	463 (4.7)	
NYHA functional class			**<0.001**
I	11055 (35.9)	2287 (32.1)	
II	11165 (36.3)	2589 (36.3)	
III	7169 (23.3)	1828 (25.6)	
IV	1394 (4.5)	432 (5.9)	
Kidney failure	7631 (18.6)	2862 (29.7)	**<0.001**
Dialysis	144 (0.4)	34 (0.4)	0.983
Diabetes mellitus	10222(25.1)	3123 (32.6)	**<0.001**
Chronic pulmonary disease	3807 (9.2)	1095 (11.2)	**<0.001**
Extracardiac arteriopathy	4500 (10.9)	1199 (12.3)	**<0.001**
Neurological dysfunction	733 (1.9)	201 (2.2)	0.057
Prior CVA	1567 (4.6)	408 (5.1)	0.075
Critical preoperative state	8383 (2.1)	239 (2.4)	0.056
Unstable angina	3497 (8.5)	1047 (10.8)	**<0.001**
Recent MI (<90 days)	13660 (33.0)	3258 (33.4)	0.469
EuroSCORE II	1.91 ± 2.32	2.94 ± 3.32	**<0.001**

Abbreviations: BMI, body mass index; LVEF, left ventricular ejection fraction; NYHA, New York heart association; CVA, cerebrovascular accident; MI, myocardial infarction; EuroSCORE, European system for cardiac operative risk evaluation.

### Operative variables

The majority of surgery was performed in a non-emergent setting (93.2%). More than 99% of patients received a LITA. All arterial grafts except the GEA were more frequently used in men as compared to women. Aortic cross-camp time per distal anastomosis was 15 minutes, and did not differ between sexes (p = 0.100) (Table 2). In the subset of the population aged 70 years or younger, similar differences between sexes were observed. As opposed to men, women received fewer arterial distal anastomoses and were less often treated with MAG.

**Table 2:** Operative variables of the general population. Mean ± standard deviation, median [range], number (percentage).

	Men (n = 41379)	Women (n = 9758)	p-value
Non-emergent surgery	38722 (93.6)	8950 (91.7)	**<0.001**
Off-pump	6711 (16.3)	1655 (17.0)	0.097
LITA	41195 (99.6)	9697 (99.4)	**0.020**
RITA	6755 (16.3)	1033 (10.6)	**<0.001**
BITA	6439 (15.8)	952 (9.9)	**<0.001**
RA	3801 (9.2)	711 (7.3)	**<0.001**
GEA	30 (0.1)	7 (0.1)	0.980
MAG	10302 (26.2)	1737 (18.8)	**<0.001**
Venous graft	30117 (72.8)	7399 (75.8)	**<0.001**
Number of arterial grafts	1.3 ± 0.5	1.2 ± 0.4	**<0.001**
Number of distal arterial anastomoses	1.8 ± 1.1	1.5 ± 0.9	**<0.001**
Number of distal venous anastomoses	1.7 ± 1.3	1.7 ± 1.3	0.075
Aortic cross-clamp time per distal anastomosis (min)*	15.0 [12.3-18.8]	15.25 [12.3-19.0]	0.100

Abbreviations: LITA, left internal thoracic artery; RITA, right internal thoracic artery; BITA, bilateral internal thoracic arteries; RA, radial artery; GEA, gastroepiploic artery; MAG, multi-arterial grafting. *Calculated for patients undergoing surgery on arrested heart, n = 33439.

### Graft choice

In the general population, all comorbidities but extracardiac arteriopathy decreased the likelihood of MAG, as did female sex (OR 0.766, 95% CI 0.718–0.818, p < 0.001). The only factor increasing the likelihood of MAG was being of average height or above (OR 1.153, CI 1.089–1.221, p < 0.001).

The population aged 70 years and younger was comprised of 19250 patients, of which 15.7% was female. MAG was used in 35.6% of men and in 26.5% of women (p < 0.001). Factors decreasing the likelihood of MAG in women were prior CVA, kidney failure, extracardiac arteriopathy, a decreased LVEF, and a higher NYHA functional class were associated with a decrease in MAG. In men, all comorbidities but neurological dysfunction decreased the likelihood of MAG use. In both sexes, MAG was more likely to be applied in non-emergent surgery and in increased body height (Table 3).

**Table 3:** Logistic regression analysis on predictors for use of multiple arterial grafts in women and in men aged 70 years and younger.

	Men (n = 16220)			Women (n = 3030)		
	OR	95% CI	p-value	OR	95% CI	p-value
Height (per cm)	1.021	1.016-1.025	**<0.001**	1.023	1.011-1.036	**<0.001**
LVEF ≥50%	Reference		**<0.001**	Reference		**0.001**
LVEF 30–49%	0.814	0.750-0.884	**<0.001**	0.700	0.551-0.889	**0.003**
LVEF <30%	0.371	0.293-0.469	**<0.001**	0.438	0.222-0.862	**0.017**
Diabetes mellitus	0.707	0.652-0.767	**<0.001**	0.922	0.766-1.109	0.389
Chronic pulmonary disease	0.780	0.682-0.893	**<0.001**	0.768	0.568-1.038	0.086
Extracardiac arteriopathy	0.725	0.638-0.823	**<0.001**	0.702	0.518-0.951	**0.022**
Neurological dysfunction	0.851	0.626-1.157	0.303	1.446	0.695-3.007	0.324
Prior CVA	0.751	0.617-0.914	**0.004**	0.541	0.315-0.929	**0.026**
Kidney failure	0.635	0.567-0.710	**<0.001**	0.571	0.452-0.722	**<0.001**
NYHA I	Reference		**<0.001**	Reference		**<0.001**
NYHA II	0.757	0.701-0.817	**<0.001**	0.600	0.493-0.730	**<0.001**
NYHA III	0.690	0.630-0.756	**<0.001**	0.635	0.508-0.793	**<0.001**
NYHA IV	0.698	0.576-0.846	**<0.001**	0.588	0.383-0.905	**0.016**
Non-emergent surgery	3.869	3.094-4.837	**<0.001**	2.422	1.507-3.892	**<0.001**
Recent MI	1.012	0.942-1.087	0.753	0.921	0.766-1.108	0.382

Abbreviations: OR, odds ratio; CI, confidence interval; LVEF, left ventricular ejection fraction; CVA, cerebrovascular accident; NYHA, New York Heart Association; MI, myocardial infarction.

### Short-term complications

Prior to propensity score matching, the incidence of DSWI, cerebrovascular complications, repeat cardiac surgery, and in-hospital mortality was higher among women (S2 Table). Following matching, baseline characteristics were well balanced (S3 Table, S4 Fig). Repeat cardiac surgery remained more frequent in women, whereas resternotomy was more common in men. No other postoperative complications differed significantly between groups (Table 4). Occurrence of DSWI after BITA versus SITA did not differ (1.0% vs 1.0%, p = 0.622).

**Table 4:** Short-term postoperative complications after propensity score matching. Number (percentage).

	Men (n = 7418)	Women (n = 7418)	p-value
DSWI	80 (1.1)	95 (1.3)	0.271
Resternotomy	343 (4.6)	228 (3.1)	**<0.001**
CVA	58 (0.8)	59 (0.8)	1.000
Vascular complication	15 (0.2)	18 (0.2)	0.742
Repeat cardiac surgery	21 (0.3)	38 (0.5)	**0.036**
In hospital mortality	69 (0.9)	82 (0.8)	0.326

Abbreviations: DSWI, deep sternal wound infection; CVA, cerebrovascular incident.

### Follow-up

#### Survival.

**Fig 1:**
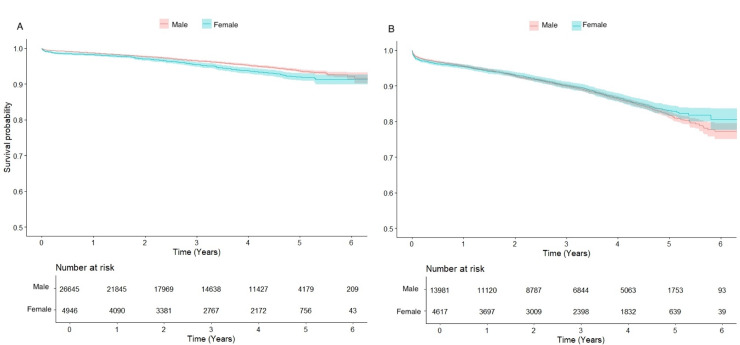
Kaplan Meier curves and number at risk for survival in years for men and women. A. Survival for the population aged 70 years and younger, p < 0.001. B. Survival for the population aged more than 70 years p = 0.410.

**Fig 2:**
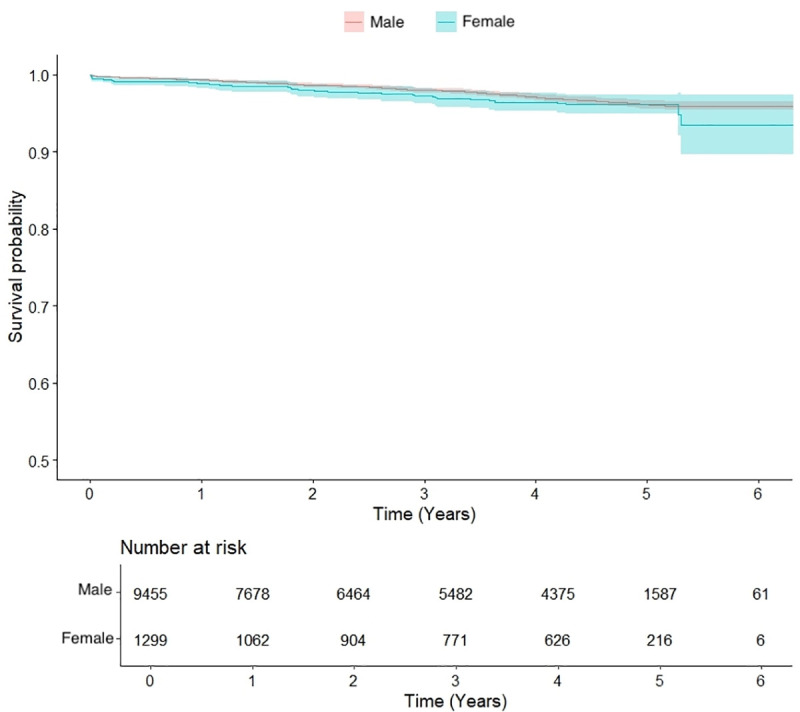
Kaplan Meier curve and number at risk for survival of men and women in years after multi-arterial grafting, in the patient population aged 70 years and younger (p = 0.231).

Median follow-up time for survival was 3.2 (1.4–4.7) years. The 1-, 3-, and 5-year overall survival rates were 97%, 94% and 89%, respectively. Although in the elderly population survival did not differ between sexes (p = 0.410), survival in women in both the general and the younger population was inferior when compared to men (p < 0.001) (Fig 1). In patients aged 70 years or younger undergoing MAG, survival of men and women was equal (p = 0.231) (Fig 2).

Cox regression survival analysis included 38987 cases. Determinants for mortality were age, unstable angina and all comorbidities. Sex was not a significant predictor for survival in multivariate analysis (Table 5). MAG was associated with a 25% mortality reduction independently of sex and age, and both age and sex did not have a significant interaction (effect modifying) effect.

**Table 5:** Multivariate Cox regression survival analysis for the overall population.

		95% confidence interval		
Variable	HR	Lower	Upper	P value
Female sex	0.954	0.867	1.050	0.334
Age (per year)	1.072	1.066	1.077	**<0.001**
Diabetes mellitus	1.539	1.418	1.669	**<0.001**
LVEF ≥ 50%	Reference			**<0.001**
LVEF 30–49%	1.626	1.491	1.775	**<0.001**
LVEF <30%	3.117	2.718	3.574	**<0.001**
Chronic pulmonary disease	1.812	1.642	2.000	**<0.001**
Extracardiac arteriopathy	1.927	1.755	2.116	**<0.001**
Neurological dysfunction	1.721	1.390	2.131	**<0.001**
Unstable angina	1.276	1.124	1.449	**<0.001**
Recent MI	1.236	1.137	1.344	**<0.001**
Prior CVA	1.246	1.069	1.453	**0.005**
Kidney failure	1.508	1.386	1.641	**<0.001**

Abbreviations: HR, hazard ratio; LVEF, left ventricular ejection fraction; MI, myocardial infarction; CVA, cerebrovascular accident.

#### Repeat revascularization.

**Fig 3:**
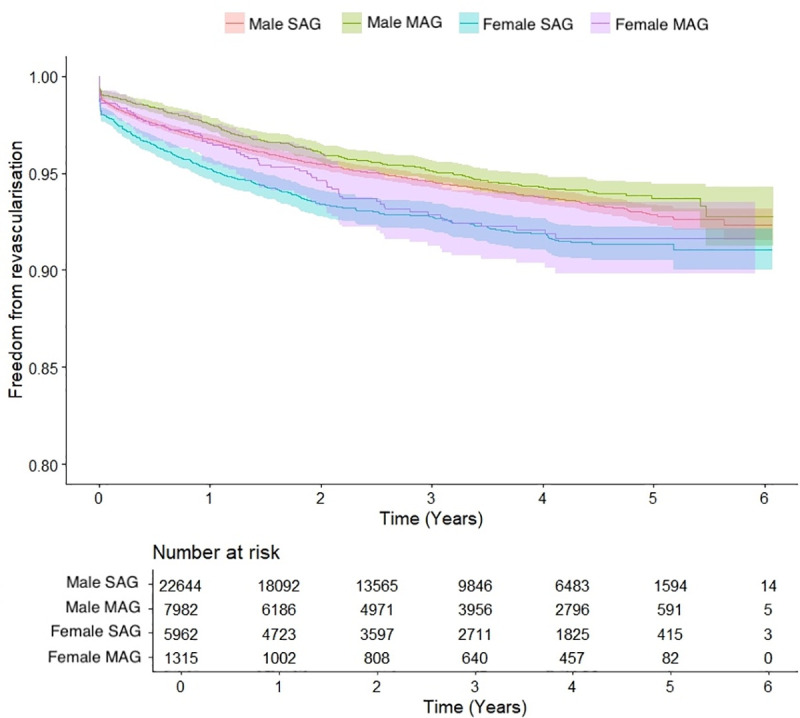
Kaplan Meier curve and number at risk for repeat revascularization in years after single- or multi-arterial grafting in men (p = 0.039) and women (p = 0.506).

During a median follow-up of 2.7 (1.2–4.3) years, a significant difference in freedom from repeat revascularization after MAG or SAG was observed in men (p = 0.039), in favor of MAG. In women, freedom from repeat revascularization did not differ after MAG or SAG (p = 0.506) (Fig 3).

One of the determinants for repeat revascularization events during follow-up was female sex (HR 1.415, 95% CI 1.273–1.572). Other variables increasing the risk of repeat revascularization were extracardiac arteriopathy (HR 1.366, 95% CI 1.202–1.553, p < 0.001), chronic pulmonary disease (HR 1.263, 95% CI 1.101–1.448, p < 0.001) and diabetes mellitus (HR 1.132, 95% CI 1.025–1.249, p = 0.014).

## Discussion

Women were of higher age and exhibited a more extensive risk profile at baseline and were less often treated with MAG compared to men in both the overall and the younger population. This combination of factors leads to an increased mortality and morbidity after CABG. The survival difference disappears in the population aged 70 years or younger after MAG, implicating the absolute survival benefit of MAG.

In line with current literature, we observed disadvantageous baseline profile in women undergoing CABG, which increases perioperative risk of death and complications [1–4,21–23]. Women were treated less often using MAG in both the general and the younger population, which does not seem to be affected by presence or absence of diabetes and chronic pulmonary disease that influence decision-making in men. A recent study by Jang and colleagues showed that equal mid-term outcomes in survival and MACCE are observed for both sexes when the vast majority of patients is treated with MAG [24]. The question arises why younger women are not treated with MAG, when they derive such a profound benefit from it, that their survival and complication rates equal that of men. Lower use of MAG in women is reportedly because they have smaller coronary arteries and graft vessels, which leads to more technically challenging surgery [25,26]. Our results confirmed the existence of this belief in the Netherlands, as greater body height was associated with increased MAG use. However, as our study and others have shown, distal anastomosis time is similar in men and women [27].

While we found that MAG was protective against repeat revascularization in men, this was not the case in women. This suggests a difference in the etiopathogenesis of graft failure between sexes. Even though women undergoing MAG were not protected from repeat revascularization, an absolute survival benefit irrespective of sex and age was demonstrated. Therefore, neither smaller vessel size nor expected difficulty of the anastomosis should be seen as reasons to forego MAG. The ROMA:women trial, an all-women cardiac surgery trial comparing SAG and MAG, will further elucidate possible effects of MAG in the female population [28].

There are some apparent limitations to this study. The NHR collects a finite number of variables, therefore there are variables influencing outcomes that were not assessed. For example, we cannot analyze incomplete revascularization as the number of diseased vessels on coronary angiography and the intended distal graft formula as described by the Heart Team are unknown. Data on planned staged or hybrid procedures are missing, which impacts the repeat revascularization variable. The influence of other postoperative factors during long-term follow up is unaccounted for as well, as women less often receive medication according to guidelines on secondary cardiovascular disease prevention and suffer more adverse events related to medication, which in turn is detrimental to their current and future health [29].

The registry provides real-world data on surgical strategy from a substantial population with hard end-points for mid-term outcome. We were able to show significant differences in revascularization strategy between men and women undergoing CABG, and the absolute survival benefit of MAG was evident. It is of great importance that we work to eliminate any subconscious selection bias for MAG, so that we can improve outcome after CABG in the future in both sexes, but especially in women.

## Conclusion

Women undergoing CABG in the Netherlands are older and suffer from more comorbidity compared to men, which leads to higher mortality during follow-up. In the younger patient population treated with MAG, survival is equal between men and women, suggesting an absolute survival benefit for women. MAG in men is associated with less repeat revascularization, which is not the case in women, a finding which deserves further examination.

## Supporting information

S1 File Definitions of postoperative complications. (PDF)

S2 Table Short-term outcome of men and women before propensity score matching. (DOCX)

S3 Table Baseline characteristics after propensity score matching between men and women. (DOCX)

S4 Fig Covariate balance before and after propensity score matching between men and women. (PDF)
