# Supplementary material for: Sex-related differences in single- and multi-arterial coronary artery bypass grafting: Insights from the Netherlands Heart Registration
Source: PLoS One. 2025 Dec 31;20(12):e0336035. doi: 10.1371/journal.pone.0336035 (PMC12755770; doi:10.1371/journal.pone.0336035)
Supplement: S1 File — (PDF) [file pone.0336035.s001.pdf]

S1 File. Definitions of postoperative complications.

DSWI: Deep sternal wound infection (mediastinitis) within 30 days. Includes muscle, sternum, mediastinum and is positive if one or more of the following criteria are present:
o Surgical drainage / refixation of the sternum for deep sternal wound infection
o Positive wound cultures.
o Antibiotic treatment for the sternal wound.
This also includes a deep sternal wound infection that occurred after the patient was discharged from the hospital in question.

Resternotomy: Reintervention due to a complication of the current intervention within 30 days. This also concerns reinterventions that were performed after the patient was discharged from the hospital in question. This concerns the first reintervention after initial closure of the thorax. Applies to all causes except opening the sternum in connection with mediastinitis or refixation of the sternum.

Major and minor vascular complications: occurrence of a vascular complication during hospitalization, diagnosis according to Valve Academic Research Consortium-2 (VARC-2) definitions, from the start of the current intervention (including perioperative vascular complications and excluding CVA).

Repeat cardiac surgery: Current intervention results in a new cardiac operation during the same admission. This also includes when a lesion to the heart is found requiring reintervention during a resternotomy, but does not include bleeding complications due to for instance a side-branch of a graft that is clipped during the resternotomy.

Second intensive care unit (ICU) stay: a second admission to the ICU after initial postoperative discharge to the ward, during the postoperative hospital stay.

Transient ischemic attack (TIA): neurologist determined that a central neurological deficit (CVA) occurred during the postoperative period, but without residual damage at the moment of discharge.

Cerebrovascular accident (CVA): permanent neurological dysfunction (excluding TIA) diagnosed by a neurologist as a result of focal ischemia of the brain, spinal cord or retina caused by an acute infarction of the neurological tissue due to thrombosis, embolism, systemic hypoperfusion or hemorrhage.
