# Supplementary material for: Sex-related differences in single- and multi-arterial coronary artery bypass grafting: Insights from the Netherlands Heart Registration
Source: PLoS One. 2025 Dec 31;20(12):e0336035. doi: 10.1371/journal.pone.0336035 (PMC12755770; doi:10.1371/journal.pone.0336035)
Supplement: S2 Table — (DOCX) [file pone.0336035.s002.docx]

S2 Table. Short-term postoperative complications of the entire population. Number (percentage).

|  | **Men (n=41379)** | **Women (n=9758)** | **p-value** |
| --- | --- | --- | --- |
| DSWI | 325 (0.9) | 119 (1.4) | **<0.001** |
| Resternotomy | 1576 (4.2) | 269 (3.0) | **<0.001** |
| Second ICU stay | 933 (2.4) | 237 (2.6) | 0.321 |
| TIA | 116 (0.3) | 41 (0.4) | **0.025** |
| CVA | 240 (0.6) | 82 (0.8) | **0.003** |
| Vascular complication | 61 (0.2) | 20 (0.2) | 0.204 |
| Repeat cardiac surgery | 132 (0.3) | 54 (0.6) | **<0.001** |
| In hospital mortality | 307 (0.7) | 128 (1.3) | **<0.001** |

Abbreviations: DSWI, deep sternal wound infection; ICU, intensive care unit; TIA, transient ischemic attack; CVA, cerebrovascular incident.
