# Supplementary material for: Sex-related differences in single- and multi-arterial coronary artery bypass grafting: Insights from the Netherlands Heart Registration
Source: PLoS One. 2025 Dec 31;20(12):e0336035. doi: 10.1371/journal.pone.0336035 (PMC12755770; doi:10.1371/journal.pone.0336035)
Supplement: S3 Table — (DOCX) [file pone.0336035.s003.docx]

S3 Table. Baseline variables after propensity score matching between men and women.

|  | Men | Women | **SMD** |
| --- | --- | --- | --- |
| Age (years) | 68.9 ± 8.8 | 68.8 ± 9.4 | -0.0033 |
| BMI (kg/m^2^) | 27.6 ± 3.9 | 27.8 ± 4.9 | 0.0344 |
| LVEF  ≥50%  30-49%  <30% | 5882 (79.3)  1327 (17.9)  209 (2.8) | 5781 (77.9)  1397 (18.8)  240 (3.2) | -0.0136  0.0042  0.0042 |
| Kidney failure | 2185 (29.5) | 2176 (29.3) | -0.0012 |
| Diabetes mellitus | 2390(32.2) | 2456 (33.1) | 0.0089 |
| Chronic pulmonary disease | 797 (10.7) | 829 (11.2) | 0.0043 |
| Extracardiac arteriopathy | 902 (12.2) | 914 (12.3) | 0.0016 |
| Neurological dysfunction | 135 (1.8) | 144 (1.9) | 0.0012 |
| Prior CVA | 349 (4.7) | 385 (5.2) | 0.0049 |
| Recent MI (<90 days) | 2469 (33.3) | 2530 (34.1) | 0.0082 |
| Procedural urgency  Elective  Urgent  Emergent  Salvation | 3797 (51.2)  3182 (42.9)  423 (5.7)  16 (0.2) | 3808 (51.3)  3141(42.3)  451 (6.1)  18 (0.2) | 0.0015  -0.0055  0.0038  0.0003 |
