# Supplementary figures and images for: Sex-related differences in single- and multi-arterial coronary artery bypass grafting: Insights from the Netherlands Heart Registration
Source: PLoS One. 2025 Dec 31;20(12):e0336035. doi: 10.1371/journal.pone.0336035 (PMC12755770; doi:10.1371/journal.pone.0336035)

# Covariate Balance

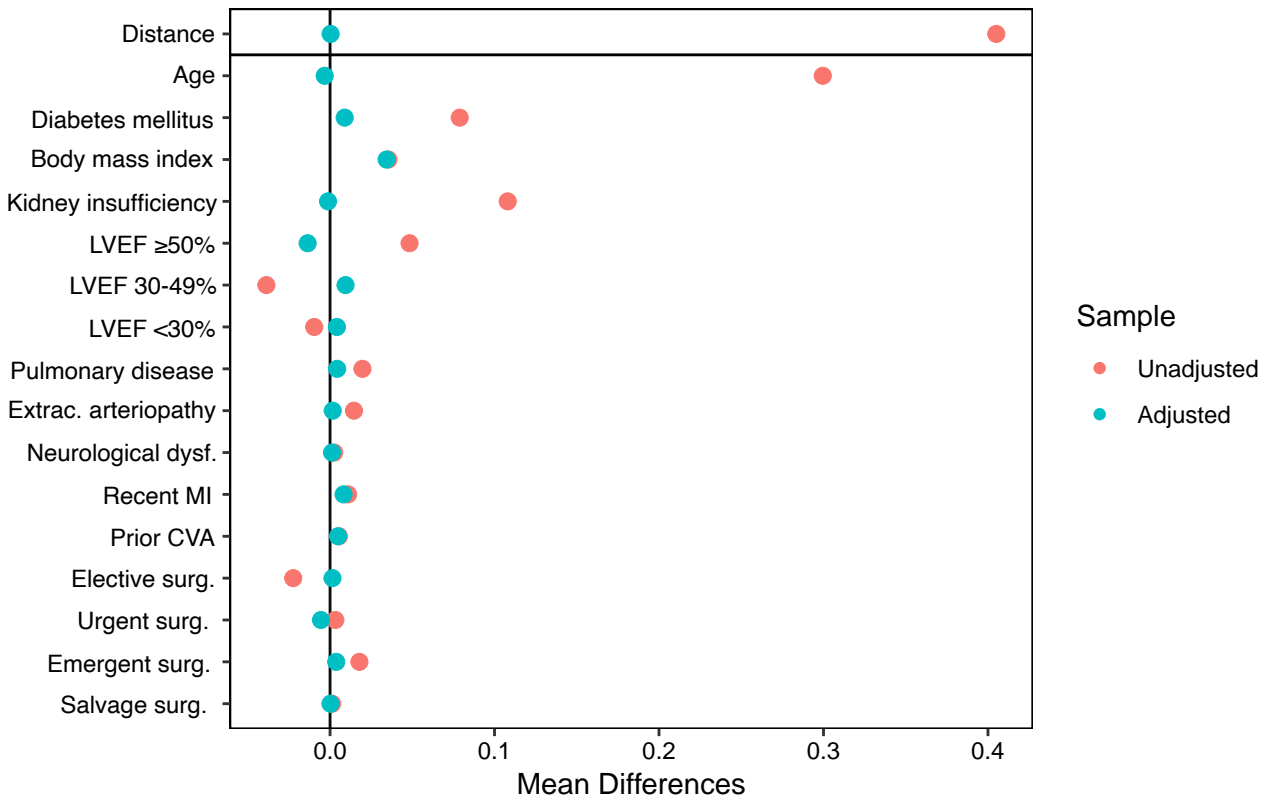

Supplement: S4 Fig — (PDF) [file pone.0336035.s004.pdf]
